# Construct validity of the Lifespan Sibling Relationship Scale measuring adult sibling relationship quality where one sibling has intellectual or developmental disabilities

**DOI:** 10.1080/20473869.2023.2301191

**Published:** 2024-01-11

**Authors:** JinJue Lin, Nikita K. Hayden, Richard P. Hastings

**Affiliations:** 1 Department of Education Studies, University of Warwick, Coventry, UK; 2 Centre for Research in Intellectual and Developmental Disabilities, University of Warwick, Coventry, UK; 3 iHuman, University of Sheffield, Sheffield, UK; 4 School of Education, University of Sheffield, Sheffield, UK

**Keywords:** Sibling relationships, Lifespan Sibling Relationship Scale, intellectual and developmental disabilities, confirmatory factor analysis, exploratory factor analysis, families

## Abstract

The Lifespan Sibling Relationship Scale (LSRS) has been validated in samples where neither sibling has intellectual or developmental disabilities. We sought to examine the construct validity of the LSRS with a sample of adult siblings of people with intellectual or developmental disabilities. Adult siblings of people with intellectual or developmental disabilities (*N* = 646) completed Adult LSRS items measuring Affect, Behavior, and Cognitions. Confirmatory Factor Analysis (CFA) and Exploratory Factor Analysis (EFA) were employed to examine the construct validity of the LSRS in this sample. The initial CFA fit for a three-factor model was inadequate (CFI = 0.86, TLI = 0.84, RMSEA = 0.10, $x^{2}\;$= 1752.97, *df* = 249). Further analyses using EFA suggested alternative two-factor and three-factor models. CFA models based on these potential factor solutions also had inadequate fit. These findings indicate that the LSRS may not be an appropriate measure of sibling relationships where one sibling has intellectual or developmental disabilities. Future research may need to utilize sibling relationship measures developed specifically for siblings where one has intellectual or developmental disabilities.

## Introduction

Sibling relationships are potentially the longest lasting relationship an individual will experience (Dunn, 2014). Siblings can influence one another’s cognitive, social, and emotional development across their lifespan (Noller, 2005). In the general population, sibling relationship quality is a predictor of mental health problems in both childhood and adulthood (Feinberg *et al.* 2012; Waldinger *et al.* 2007). Research about siblings of people with intellectual or developmental disabilities typically position siblings as an important source of care, support, advocacy, and friendship for one another (Hayden *et al.* 2023a; Hayden and Hastings, 2022). A survey of 1,160 adult siblings found that the majority of siblings reported that they trusted, respected, were fair towards, and felt affection for their disabled siblings (Hodapp *et al.* 2010). A range of factors have been found to be associated with more positive sibling relationships, such as the gender of the non-disabled sibling (Orsmond *et al.* 2009; Hodapp *et al.* 2010), the behaviours and the disability type of the disabled sibling (Orsmond *et al.* 2009; Orsmond and Seltzer, 2007; Hodapp and Urbano, 2007), and familial factors, such as the number of non-disabled siblings (Hodapp *et al.* 2010). A study by Burbidge and Minnes (2014) found that siblings without a developmental disability had more frequent contact with, and more positive feelings about, their sibling *with* a developmental disability in comparison to their feelings and contact with their sibling *without* a developmental disability. Another study found that adult siblings of people with intellectual or developmental disabilities had less positive attitudes towards their sibling relationships (measured using the Lifespan Sibling Relationship Scale (LSRS; Riggio, 2000)) compared to adult siblings of people without intellectual or developmental disabilities (Sommantico *et al.* 2020a).

The reason why fostering sibling relationships may be particularly important in this population is that research suggests that there is an association between more “positive” sibling relationships, and siblings taking on a care or support role for their siblings with intellectual or developmental disabilities (Burke *et al.* 2012; Lee and Burke, 2018). Hayden *et al.* (2022) have argued that those interested in the future care and wellbeing of disabled adults should perhaps be interested in ways of improving and fostering the relationship between siblings when one sibling has intellectual or developmental disabilities (Hayden *et al.* 2022: 6). There are important social care^1^ implications related to fostering the sibling relationships of this population. Understanding sibling relationships where one sibling has intellectual or developmental disabilities is an important step in supporting and fostering these siblings’ relationships.

Identifying appropriate ways of measuring sibling relationships where one sibling has intellectual or developmental disabilities is an important step in understanding and fostering these sibling relationships. However, sibling relationships are complex (Davies, 2023). Sibling relationships are private, dynamic, lifelong, intense, unchosen, and ever changing. Although instruments have been developed for the general population to measure sibling relationship quality in adults and children, there is little research on how well these measures capture the experiences of siblings where one sibling has intellectual or developmental disabilities. It is important that we examine existing measures of sibling relationship quality, so as not to “reinvent the wheel”: as there are likely to be existing measures that may work (with or without adaptations) for this population.

In previous research, construct validity for a general measure of sibling relationship quality used with families of children with intellectual or developmental disabilities was found to be inadequate (Hayden *et al.* 2023a). Participating families also shared that they found it difficult and frustrating to complete these items, as they did not work well when the sibling with an intellectual disability was minimally verbal or when the siblings did not interact often. There are various reasons why we might theorise that existing measures of sibling relationship quality designed for the general population, may not be the best instruments to use for sibling dyads where one sibling has intellectual or developmental disabilities: First, there is a lot of complexity and variability in sibling relationships for this population; Second, it is hard to answer a lot of these items if you are reporting on a sibling who is minimally verbal; Third, siblings often tell us that the reciprocity in their relationship is not always obvious to, or well understood by, outsiders; Fourth, that siblings may interpret experiences such as rivalry or parental differential treatment very differently, especially in adulthood, when one sibling has additional support needs. Given these complexities and uncertainties, along with the importance of sibling relationship quality in this population, it is important to investigate the utility of general sibling relationship measures in this population.

### The Lifespan Sibling Relationship Scale

The Lifespan Sibling Relationship Scale is a self-report measure used to measure adults’ attitudes towards their sibling relationships in both childhood (retrospectively) and adulthood (Riggio, 2000). The LSRS is an English language scale composed of six subscales: Adult Affect, Adult Behavior, Adult Cognitions, Child Affect, Child Behavior, and Child Cognitions. Each subscale consists of eight items. The higher the score, the more “positive” they are about their sibling relationship. Respondents are required to complete the scale with only one sibling relationship in mind, and all 48 statements are rated using a five-point Likert scale (Riggio, 2000). A total of 711 undergraduate and graduate students (mean age = 23.5) participated in Riggio’s (2000) study. The LSRS, although validated with a young adult sample, was conceptually developed to measure siblings’ relationships over the lifespan. Good psychometric properties were reported with high internal consistency for all six LSRS subscales and the total score (Riggio, 2000). The Cronbach’s alpha for the total LSRS score was .96. The six LSRS subscales also scored high coefficient alphas (Adult Affect = 0.91, Adult Behavior = 0.87, Adult Cognitions = 0.91, Child Affect = 0.89, Child Behavior = 0.84, and Child Cognitions = 0.88). In addition, the LSRS has been adapted and successfully validated in other languages, including Korean, Turkish, Italian, and English (Jeong *et al.* 2013; Öz, 2015; Sommantico *et al.* 2017; Cilalı, Erdur-Baker and Bugay, 2019; Gungordu *et al.* 2021).

### Research aims and objectives

Although the LSRS has been used in intellectual or developmental disabilities sibling research, it has not yet been validated as a measure of sibling relationships where one sibling has intellectual or developmental disabilities. The main aim of the current study, therefore, was to examine the construct validity of the LSRS in measuring sibling relationships with a sample of adult siblings of people with intellectual or developmental disabilities, using confirmatory factor analysis and exploratory factor analysis.

## Methods

### Participants

The initial sample consisted of 911 participants who took part in an online survey about being an adult sibling of someone with intellectual or developmental disabilities (Hayden *et al.* 2023b). We included in the current analyses 646 participants where both the participant and the brother/sister are adults (defined as ≥ 16) and completed all 24 LSRS items. Participants’ mean age was 35.25 (*SD*** **=** **12.53, Range = 18 to 73 years); 89 participants identified as “male,” 553 identified as “female,” and four selected “gender not listed.” The mean age of their brothers and sisters with intellectual or developmental disabilities (*N*** **=** **644, 2 missing) was 33.76 years (*SD*** **=** **12.78, Range = 16 to 83 years). Siblings reported on the gender of their brothers and sisters with intellectual or developmental disabilities. 385 siblings with intellectual or developmental disabilities were identified as “male,” 252 were identified as “female,” seven selected “gender not listed” and two were reported missing.

Participants were asked to select conditions/labels that have been used to describe their siblings with intellectual or developmental disabilities. Multiple items could be selected and participants were instructed to “select all that apply” if their siblings had multiple conditions. In total, 59.3% (*n*** **=** **383) reported their brother/sister had an intellectual disability, 46.7% (*n*** **=** **302) had autism, 35.6% (*n*** **=** **230) had Down syndrome, 9.4% (*n*** **=** **61) had cerebral palsy, and 16.1% (*n*** **=** **104) had “other” genetic syndromes. These diagnoses were reported by the sibling. Tables 1 and 2 provided details on participants’ and their adult siblings’ demographic characteristics.

**Table 1.** Demographic characteristics of participants.

Variable	Category	N	%
Ethnicity	Asian/Asian British: Indian	3	0.5
	Asian/Asian British: Pakistani	1	0.2
	Black/African/Black British: African	1	0.2
	Black/African/Black British: Caribbean	1	0.2
	Black not listed	1	0.2
	Mixed/multiple ethnic groups: White and Black Caribbean	1	0.2
	Mixed/multiple ethnic groups: White and Black African	1	0.2
	Mixed/multiple ethnic groups: White and Asian	3	0.5
	Mixed not listed	4	0.6
	Ethnicity not listed	2	0.3
	White: English/Welsh/Scottish/Northern Irish/British	600	92.9
	White: Irish	9	1.4
	White not listed	15	2.3
	Any other ethnic background	4	0.6
Highest level of educational qualification	L3 qualification and below	128	19.8
	Higher Education but below degree level (i.e. foundation degree diploma, higher apprenticeship)	92	14.2
	Degree (e.g. BA, BSc, MA)	425	65.8
	Missing	1	0.2
Caregiver*	No	295	45.7
	Yes	350	54.2
	Missing	1	0.2
Equivalized income**	Above poverty line	181	28.0
	Below poverty line	133	20.6
	Missing	332	51.4
IMD Decile 1 = Most deprivation 10 = Least deprivation	1	33	5.1
	2	38	5.9
	3	58	9.0
	4	47	7.3
	5	52	8.0
	6	65	10.1
	7	64	9.9
	8	71	11.0
	9	62	9.6
	10	80	12.4
	Missing	76	11.8

* We used the NHS definition of a ‘carer’ (NHS, 2022): ‘A carer is anyone, including children and adults, who looks after a family member, partner or friend who needs help because of their illness, frailty, disability, a mental health problem or an addiction and cannot cope without their support. The care they give is unpaid’.

** Equivalized income is defined as above or below 60% of UK median household income.

**Table 2.** Demographic characteristics of participants’ siblings with IDD.

Variable	Category	N	%
Other conditions (additionally)	Visual impairment	195	30.2
	Hearing impairment	133	20.6
	Mobility problem	268	41.5
	Physical health problems	319	49.4
	Epilepsy/seizures	169	26.2

### Measures

Three adult subscales from Riggio’s (2000) LSRS were included in this study. These measured: Adult Affect, Adult Behavior, and Adult Cognitions. We did not include the child items in the Adult Sibling Survey in order to reduce participant burden, improve survey completion, and because we were empirically interested in siblings’ assessment of their current sibling relationships. Adult Affect measured siblings’ emotions towards their sibling and their sibling relationships, for example, “I enjoy my relationship with my sibling.” Adult Behavior assessed siblings’ interactions with their sibling and the perceived positivity of their interactions, such as “I like to spend time with my sibling.” Adult Cognitions evaluates siblings’ beliefs about their sibling and their sibling relationship, for example: “My sibling is very important in my life” (Riggio, 2000). A total of 24 items were rated using a five-point scale (1 = Strongly disagree, 2 = Disagree, 3 = Neither agree nor disagree, 4 = Agree, and 5 = Strongly agree).

### Procedure and study design

The study was designed in collaboration with UK charity Sibs, a national sibling disability non-profit organisation. Sibs also took the lead on recruitment to the study *via* their mailing list, their social media networks, and through their connections with other disability and carer non-profit organisations. Full ethical approval was granted by the Humanities and Social Sciences Research Ethics Committee (HSSREC 137/18-19) at the University of Warwick. Participants were recruited using convenience and snowball sampling strategies. The online survey was hosted on Qualtrics™. A Qualtrics link was shared *via* various non-profit organisations’ newsletters and across social media (e.g. Twitter, Facebook). The Qualtrics link took participants to a landing page which included the study information sheet. Participants had to agree to a range of statements confirming eligibility (i.e. the consent form) to proceed to the survey questions. Participants were encouraged to share the survey with other adult siblings that they knew. The inclusion criteria for participants were they had to: (1) be aged 18 years or over; (2) have a brother or a sister with an intellectual or developmental disability (for the original study, of any age); (3) reside in the UK; and (4) be willing to take part in the research.

### Analysis procedure

All analyses were performed using AMOS and SPSS 26. First, Confirmatory Factor Analysis (CFA) was performed using Maximum Likelihood Estimation with the full dataset to provide a baseline model fit of the three LSRS subscales. CFA is a hypothesis driven approach which examines the nature of, and relations among variables related to latent constructs (Jackson *et al.* 2009). It is a popular analytical tool used for developing and refining measurement instruments and assessing construct validity (Brown, 2015). Three model fit indices were used to assess all CFA models: Comparative Fit Index (CFI; ≥ 0.95), Tucker-Lewis Index (TLI; ≥ 0.95), and Root Mean Square Error of Approximation (RMSEA; ≤ 0.06). These cut-off levels were recommended by Hu and Bentler (1999). However, the model fit for the LSRS in this sample (see Results) was not adequate.

Therefore, further analyses were conducted to explore ways of improving the model using exploratory factor analysis (EFA). EFA is an exploratory method used to generate theory and therefore help us identify the best structure for the LSRS items for this specific population (Henson and Roberts, 2006). We used SPSS to divide the dataset randomly into two subsets, where approximately 50% of the random cases were filtered and allocated to each sample. Sample One consisted of 319 participants, and Sample Two consisted of 327 participants. The EFA was carried out using Sample One. Drawing on the findings of the EFA used on Sample One, we tested new models on Sample Two using CFA again to assess the validity of the new constructs identified using EFA. Adjustments were made to improve model fit where factor loadings were <0.6 in two ways. First, items were parcelled. Parcelling potentially stabilizes parameter estimates, improves model fit, and offers psychometric benefits (Matsunaga, 2008). We used the standardised regression weights table from the preceding CFA model to inform which items have a regression weight value below 0.6. Each item was aggregated with other higher weighting items within the same factor to create new “parcels” or units. The created parcels replaced the original items as new variables for the target latent construct. Second, low-loading items were dropped from the model. A forward entry approach was used to explore these models iteratively.

## Results

### Full sample: confirmatory factor analysis

The initial CFA tested the LSRS constructs as defined by Riggio (2000) as three, distinct latent constructs, each with eight items: Adult Affect, Adult Behavior, and Adult Cognitions. The original LSRS factor model had an inadequate model fit (CFI = 0.86, TLI = 0.84, RMSEA = 0.10, $x^{2}$ = 1752.97, *df*** **=** **249). We then examined a single construct model by including all 24 items on a single latent construct and again, the model fit was inadequate (CFI = 0.76, TLI = 0.74, RMSEA = 0.12, $x^{2}$ = 2741.08, *df*** **=** **252). Parcelling techniques were then used to further examine whether we could improve the model fit. However, no models provided adequate fit to these data (see Table 3).

**Table 3.** Model fit indices comparison.

	Model #	CFI	TLI	RMSEA	Chi-square ($x^{2}$)	Degrees of Freedom (*df*)
LSRS (Original)	Original	0.86	0.84	0.10	1752.97	249
Single Factor	24 Items	0.76	0.74	0.12	2741.08	252
	Model 1A	0.87	0.84	0.14	1055.40	77
	Model 1B	0.87	0.85	0.13	863.54	77
	Model 1C	0.89	0.88	0.12	819.72	77

The initial single-factor model indicated ten items weighing below 0.6. Model 1A dropped all these ten items. Model 1B parcelled the ten items with higher loading items; Model 1C dropped Items 6, 10, 13, 14 and 16 value < 0.5 and parcelled the other five.

### Exploratory factor analysis

Data from Sub-Sample One were used to examine alternative model configurations using EFA. During the initial inspection of the data, the KMO value obtained was 0.946, indicating that the sample was adequate to proceed with EFA. However, the correlation matrix suggested that item 6 (“they frequently make me very angry”), item 10 (“I call them on the telephone frequently”), item 13 (“I never talk about my problems with them”) and item 16 (“they talk to me about personal problems”) had a total of 12 or more correlations below 0.3, which accounted for 50 per cent of associations. Hence, these four items were removed before proceeding any further with the EFA. Next, factors were extracted using principal axis factoring. The eigenvalues greater-than-one rule suggested a three-factor model, whilst the elbow on the scree plot indicated a two-factor model. Therefore, both two- and three-factor models were explored. Then, oblique (direct oblimin) rotation was conducted. The pattern matrix on the two-factor model showed that items 23 (“I know I am one of their best friends”) and 24 (“they are proud of me”) had a loading value below 0.4 (prior to rounding up to two-digits figure) and were thus removed. All other items satisfied the rule with a loading factor above 0.4 on the primary factor and below 0.4 on alternative factors. Thus, a new list of factors and items was produced for the two-factor and three-factor models – see Table 4.

**Table 4.** Pattern matrix and factor loadings.

No	Brief Version of Item	Two-Factor		Three-Factor			
		1	2	1	2	3	
1.	They make me happy.	**0.85**	0.02	**0.87**	0.07	−0.05	
2.	I value their feelings.	**0.77**	−0.07	**0.69**	−0.04	0.09	
3.	I enjoy our sibling relationship.	**0.83**	0.07	**0.88**	0.12	−0.08	
4.	I feel proud of them.	**0.85**	−0.07	**0.75**	−0.05	0.14	
5.	We have fun together.	**0.57**	0.35	**0.64**	0.40	−0.10	
7.	I admire them.	**0.64**	0.11	**0.52**	0.10	0.20	
8.	I enjoy spending time with them.	**0.83**	0.04	**0.87**	0.10	−0.08	
9.	We currently spend a lot of time together.	0.13	**0.51**	0.14	**0.50**	0.02	
11.	We share secrets.	−0.12	**0.76**	−0.12	**0.71**	0.07	
12.	We do things together.	0.09	**0.75**	0.10	**0.72**	0.05	
14.	We borrow things from each other.	−0.14	**0.74**	−0.11	**0.72**	0.00	
15.	We ‘hang out’ with each other.	0.15	**0.68**	0.16	**0.67**	0.02	
17.	They are a good friend.	0.27	**0.63**	0.25	**0.62**	0.08	
18.	They are important in my life.	**0.85**	−0.13	**0.67**	−0.14	0.27	
19.	We are not very close.	**0.57**	0.26	**0.45**	0.23	0.21	
20.	They are one of my best friends.	0.32	**0.58**	0.29	**0.56**	0.10	
21.	We have a lot in common.	0.14	**0.56**	0.13	**0.54**	0.06	
22.	I believe I am important to them.	**0.54**	0.09	0.16	−0.07	**0.71**	
23.	I know I am one of their best friends.	**0.40**	0.35	−0.08	0.18	**0.86**	
24.	They are proud of me.	**0.32**	0.32	0.04	0.22	**0.51**	

Note. Items 6 (‘they frequently make me very angry’), 10 (‘I call them on the telephone frequently’), 13 (‘I never talk about my problems with them’) and 16 (‘they talk to me about personal problems’), although included in our CFA, were not included in our EFA, due to low factor loadings.

### Two-factor model

Using Sample Two data, the two-factor model consisted of 18 items in total. The EFA identified two potential constructs from the LSRS under this model. The authorship team discussed the items included in both EFA-identified constructs. We considered how each construct made conceptual sense to be grouped together in the way that the EFA had identified and then labelled each construct. Factor One consisted of 10 items which we described as “my feelings about my sibling” and Factor Two consisted of eight items which we summarised as “shared interests with my sibling.” The initial two-factor model appeared to have a better fit than the previously tested CFA models (CFI = 0.90, TLI = 0.88, RMSEA = 0.10, $x^{2}$ = 609.78, *df*** **=** **134). However, this model fit was still not sufficient (i.e. it did not meet the cut-off values for the goodness of fit indices). Again, three alternative models were examined. The standardised regression weights highlighted item 22 (0.59) in Factor One and items 11 (0.60) and 14 (0.52) in Factor Two (loadings <0.6 prior to rounding up to two-digits figure). In Model 2A, we dropped all three items. In Model 2B we parcelled all three items and in Model 2C we parcelled items 22 and 11 and dropped item 14 because it had the lowest value. Item 22 was parcelled with item 3 (0.93), item 11 with item 20 (0.84) and item 14 with item 12 (0.85). Parcelled items were selected from the respective factors. All alternative models showed some increase in CFI and TLI values, indicating small improvements in the model fit. The RMSEA values also increased for Models 2A and 2C, but it remained the same for Model 2B (see Table 5). Therefore, Model 2B demonstrated the best fit so far.

**Table 5.** All model fit indices comparison.

	Model #	CFI	TLI	RMSEA	$x^{2}$	*df*	Model Constructs and Items
LSRS	Original	.86	.84	.10	1752.97	249	*Factor 1*: Items 1-8; *Factor 2*: Items 9-16; *Factor 3*: Items 17-24
Single Factor	24 Items	.76	.74	.12	2741.08	252	*Factor 1*: Items 1-24
	Model 1A	.87	.84	.14	1055.40	77	*Factor 1*: Items 1-5, 7-8, 12, 15, 17-20, 23
	Model 1B	.87	.85	.13	863.54	77	*Factor 1*: Items 2, 15, 18, 23; Parcelled: 6, 9-11, 13-14, 16, 21-22, 24
	Model 1C	.89	.88	.12	819.72	77	*Factor 1*: Items 2, 4, 7, 12, 15, 18-20, 23; Parcelled: 9, 11, 21-22, 24
Two- Factor	Model 2	.90	.88	.10	609.78	134	*Factor 1:* Items 1-5, 7-8, 18-19, 22; *​​Factor 2*: Items 9, 11-12, 14-15, 17, 20-21
	Model 2A	.91	.89	.12	490.81	89	*Factor 1*: Items 1-5, 7-8, 18-19; *Factor 2*: Items 9, 12, 15, 17, 20-21
	Model 2B	.93	.92	.10	369.75	89	*Factor 1*: Items 1-2, 4-5, 7-8, 18-19; Parcelled: 22; *Factor 2*: Items 9, 15, 17, 21; Parcelled: 11, 14
	Model 2C	.91	.90	.11	447.52	89	*Factor 1*: Items 1-2, 4-5, 7-8, 18-19; Parcelled: 22; *Factor 2*: Items 9, 12, 15, 17, 21; Parcelled: 11
Three- Factor	Model 3	.89	.87	.10	743.42	167	*Factor 1*: Items 1-5, 7-8, 18-19; *Factor 2*: Items 9, 11-12, 14-15, 17, 20-21; *Factor 3*: Items 22-24
	Model 3A	.89	.87	.12	619.86	116	*Factor 1*: Items 1-5, 7-8, 18-19; *Factor 2*: Items 9, 12, 15, 17, 20-21; *Factor 3*: Items 22-23
	Model 3B	.92	.90	.10	498.87	116	*Factor 1*: Items 1-5, 7-8, 18-19; *Factor 2*: Items 9, 15, 17, 21; Parcelled: 11, 14; *Factor 3*: Item 22; Parcelled: 24
	Model 3C	.90	.88	.11	579.18	116	*Factor 1*: Items 1-5, 7-8, 18-19; *Factor 2*: Items 9, 12, 15, 17, 21; Parcelled: 11; *Factor 3*: Item 22; Parcelled: 24

Model 2 highlighted items 11, 14 and 22 with loadings < 0.6. Model 2A dropped all three items. Model 2B parcelled all three items in each respective factor. Model 2C parcelled Items 11 and 22, with higher value, and dropped Item 14.

Model 3 indicated items 11, 14 and 24 loadings < 0.6. Model 3A dropped all items. Model 3B parcelled all items in each respective factor. Model 3C parcelled higher weighting items (Items 11 and 24) and dropped Item 14.

### Three-factor model

The three-factor model contained a total of 20 items and three potential constructs identified from the LSRS. Again, our team discussed the items loaded under each EFA-identified construct to identify any changes and labelled them. In this model, Factor One consisted of nine items, which we understood as “my feelings about my sibling.” Factor Two consisted of eight items which we summarised as “shared interests with my sibling.” Factor Three consisted of three items, which we summarised as “my sibling’s perception of me.” The initial three-factor model appeared to have a better fit than the original three-factor CFA model (CFI = 0.89, TLI = 0.87, RMSEA = 0.10, $x^{2}\;$= 743.42, *df*** **=** **167). However, the model fit was still inadequate, and the recommended cut-off values were not met. Once again, three alternative models were explored. The standardised regression weights pointed out items 11 (0.59) and 14 (0.51) in Factor Two and item 24 (0.60) in Factor Three – again, prior to rounding up to two-digits figure. In Model 3A we dropped all three items. In Model 3B we parcelled all three items in each respective factor. In Model 3C we parcelled items 11 and 24 and dropped item 14 with the lowest loading value. None of the alternative models had adequate fit (see Table 5).

### Comparing model fit indices

A total of 13 CFA models were explored, including the original LSRS model, single-factor models, two-factor models, three-factor models and all the alternative models obtained from using the parcelling technique. Table 5 presents a comparison of the model fit indices for all CFA models. The initial single latent construct did not improve the model fit. However, all other CFA models showed improvements to the model fit, despite poorer RMSEA values. Model 2B can be argued to be the best model fit with increased CFI and TLI values closer to 0.95 and no difference for the RMSEA. Despite the improvements, the CFI, TLI and RMSEA indices still did not reach the cut-off point to be considered a “good” model. As such, none of the CFA models explored were a sufficient fit for the current LSRS data.

## Discussion

Overall, our findings suggest that the LSRS may lack construct validity as a measure of adult sibling relationship quality where one sibling has intellectual or developmental disabilities. The LSRS scale was designed with a sample of adults where neither sibling had intellectual or developmental disabilities. A question, therefore, arises about why the LSRS scale may not have performed well with a sample where one sibling had intellectual or developmental disabilities. In the sibling disability field, relatively little is known about the perspectives of the disabled sibling (Meltzer and Kramer, 2016; Richardson and Jordan, 2017), especially for siblings with a severe-profound intellectual disability. Some non-disabled siblings would have had difficulties completing the LSRS about their relationship with their sibling with intellectual or developmental disabilities. For example, items 13 and 16 in the LSRS loaded poorly to the Adult Behavior construct during the EFA, which described shared behaviours where siblings talk to each other. These statements do not sufficiently measure and reflect sibling relationships if the sibling with intellectual or developmental disabilities is, for example, minimally verbal and communicates non-verbally. We suggest, therefore, that the items from the three LSRS adult subscales may not be the most appropriate for this population.

The Adult Behavior subscale included items related to siblings spending time with their brothers or sisters. However, the severity of the disability may impact the shared behaviour between siblings (Rossetti *et al.* 2020). We found that statements such as “I often call them on the telephone” (item 10), “I never talk about my problems with my sibling” (item 13) and “my sibling talks to me about personal problems” (item 16) loaded poorly onto the Adult Behavior construct. These statements do not sufficiently measure and reflect sibling relationships if the sibling with intellectual or developmental disabilities is, for example, minimally verbal and communicates non-verbally; or if they are unable to use telephones without support from other people. Items for sibling relationships where one has intellectual or developmental disabilities could instead focus on time spent together, rather than time spent communicating verbally. Some siblings may also find that care is an important part of their sibling relationship. Therefore, having a separate sibling carer construct, or a separate sibling carer measure, may also be important additions.

In the Adult Cognitions subscale, item 23 (“I know I am one of their best friends”) was removed from the three-factor model due to low loading value. The existing literature suggests that siblings where one has intellectual or developmental disabilities may have less contact than other siblings (Rossetti *et al.* 2020). However, the importance of mutual support between these siblings should not be underestimated (Chase and McGill, 2019). Sibling relationships where one has intellectual or developmental disabilities have been found to be reciprocal (Kramer *et al.* 2013; Rossetti *et al.* 2020). Thus, additional constructs could be added to reflect on the complexity of their sibling relationships (e.g. “my sibling gives back or supports me in their own way” or “my sibling and I provide each other with mutual support”). These constructs could better capture the reciprocity in a sibling relationship where one has intellectual or developmental disabilities.

### Limitations and future research directions

In terms of limitations, our sample was recruited using convenience sampling, and our sample was non-representative and biased, as most of our sample was white women and the sample was disproportionately young adults. We also did not examine the impact of cultural influences on measuring sibling relationship quality in this population, which is an important area to further understand (Lee *et al.* 2021). We also only included the adult sibling items of the LSRS, removing the retrospective child sibling items. We designed the survey in this way to reduce participant burden and we chose to concentrate on adults’ perspectives of their current sibling relationships. Therefore, further testing of the LSRS in this sample may be warranted.

In the future, researchers may need to develop and utilize measures of sibling relationship quality targeting siblings of people with intellectual or developmental disabilities as a specific group. Sommantico *et al.* (2020b) have recently developed the Siblings’ Experience Quality Scale (SEQS). SEQS is a self-report instrument, with good psychometric properties, assessing emotional, behavioural, and cognitive experiences of adult siblings of people with disabilities (including intellectual disability), mental health conditions, and chronic health conditions (Sommantico *et al.* 2020b). The Italian version of the SEQS has demonstrated its suitability in assessing sibling relationships where one sibling is disabled. An English language version of the SEQS could be tested in future with an intellectual and developmental disabilities-only sample (or by disaggregating the data based on specific conditions). There is also an urgent research need to identify appropriate methods to include the perspectives of people with intellectual or developmental disabilities about their sibling relationships. Although this study has focused on adult siblings, there is a need to also consider how we examine sibling relationships where one has intellectual or developmental disabilities in childhood. The understanding of cultural influences on measuring sibling relationships in this population is also an important area of future research.

## Conclusion

Overall, our findings indicate that the use of Riggio’s (2000) LSRS with siblings of people with intellectual or developmental disabilities may lack construct validity as a measure of sibling relationship quality in this sample and should be used currently with caution. Alternative measures of sibling relationship quality in this sample should be further tested (e.g. the SEQS) and/or developed. Sibling relationship quality has been associated with adult siblings’ future caregiving plans for their brothers and sisters with intellectual and developmental disabilities (Burke *et al.* 2012). From a practice perspective, therefore, it will be valuable to be able to understand sibling relationships and how to enhance sibling relationship quality for sibling pairs where one has intellectual or developmental disabilities.

## Data Availability

There is no data set associated with this submission.

## Note

1 Social care refers to welfare provision for children and adults, such as social services, provision for personal care, protective services, and social support.
